# Genome Sequences of Avian Pathogenic *Escherichia coli* Strains Isolated from Brazilian Commercial Poultry

**DOI:** 10.1128/genomeA.00110-13

**Published:** 2013-03-21

**Authors:** Thaís Cabrera Galvão Rojas, Renato Pariz Maluta, Lucas Pedersen Parizzi, Luciano Vieira Koenigkan, Jian Yang, Jun Yu, Gonçalo Amarante Guimarães Pereira, Wanderley Dias da Silveira

**Affiliations:** a Department of Genetics, Evolution, and Bioagents, Institute of Biology, University of Campinas (UNICAMP), Campinas, São Paulo, Brazil; b Embrapa Informática Agropecuária, Campinas, São Paulo, Brazil; c MOH Key Laboratory of Systems Biology of Pathogens, Institute of Pathogen Biology, Chinese Academy of Medical Sciences & Peking Union Medical College, Beijing, China; d Strathclyde Institutes of Pharmacy and Biomedical Sciences, University of Strathclyde, Glasgow, United Kingdom

## Abstract

Avian pathogenic *Escherichia coli* (APEC) infections are responsible for significant losses in the poultry industry worldwide. The disease might present as different local infections or as septicemia. Here, we present the draft genome sequences of three Brazilian APEC strains isolated from different kinds of infections. The availability of these APEC genome sequences is important for gaining a thorough understanding of the genomic features of *E. coli*, particularly those of this pathotype.

## GENOME ANNOUNCEMENT

*Escherichia coli* strains are Gram-negative enterobacteria that are part of the normal intestinal microbiota of mammals and birds. Most *E. coli* strains are commensal, but subsets of these bacteria have acquired the capacity to cause intestinal and extraintestinal diseases in humans and animals (1, 2). *E. coli* strains responsible for bird diseases are collectively named avian pathogenic *E. coli* (APEC), and the infection due to APEC is known as colibacillosis (2, 3).

APEC infections are responsible for significant losses in the poultry industry worldwide due to high mortality, carcass condemnation, decrease of egg production, and other losses in productivity. The disease might present as a local infection, such as perihepatitis, pericarditis, peritonitis, salpingitis, cellulitis, omphalitis, air sacculitis, or swollen head syndrome, or as septicemia (4, 5).

Three APEC strains were sequenced. *E. coli* strain SEPT362 (OR:H10) was isolated from the liver of a laying hen with clinical signs of septicemia. *E. coli* strain S17 (O113:H4) was also isolated from the liver, but from a broiler chick with septicemia. *E. coli* strain O08 (O38:H10) was obtained from the yolk of the abdomen of a diseased 1-day-old chick.

The genomes were sequenced using 454 Life Sciences technology (6). The read assembly was performed with Genome Sequencer (GS) *de novo* Assembler version 2.5.3, and the resulting contigs were ordered with the PROmer application version 3.0 (7) and mapped “one for one” from the show-tiling option, using APEC O1 (available in the GenBank database accession no. CP000468.1) as a reference genome.

The genome assembly of the 3 strains resulted only in draft assemblies with high coverage, which should represent most of the functional annotated genes and allow for comparative studies using these genomes (8). Genome sequencing for the strains O08, S17, and SEPT362 resulted in 301,852, 342,817, and 305,381 reads, with average read sizes of 445, 369, and 345 bp, and coverages of 26×, 25×, and 21×, respectively. The total sizes of the assemblies were 5,084,722, 4,591,470, and 5,279,952 bp, resulting in 160, 187, and 173 contigs for the strains O08, S17, and SEPT362, respectively.

APEC genome sequences were annotated by the NCBI Prokaryotic Genomes Automatic Annotation Pipeline (PGAAP) (http://www.ncbi.nlm.nih.gov/genomes/static/Pipeline.html). The numbers of annotated genes were 4,908, 4,463, and 5,151 for O08, S17, and SEPT362 strains, respectively.

The availability of several APEC genome sequences (9, 10, 11) permits the comparison of genome contents between APEC and other pathogenic and nonpathogenic *E. coli* strains, which might help us understand the evolutionary processes involved in the shaping of the phenotypes of different pathotypes. A detailed study of these genomes and those of other available *E. coli* strains will be reported soon.

### Nucleotide sequence accession numbers. 

These GenBank Whole Genome Shotgun projects have been deposited at DDBJ/EMBL/GenBank under the accession no. AOGM00000000, AOGN00000000, and AOGL00000000 for the O08, S17, and SEPT362 strains, respectively. The versions described in this paper are the first versions, accession no. AOGM01000000, AOGN01000000, and AOGL01000000 for strains O08, S17, and SEPT362, respectively.
